# Naval Medical Appointments

**Published:** 1860-07

**Authors:** 


NAVAL MEDICAL APPOINTMENTS.—The Naval Medical Board of Examiners adjourned in this city on the fifth of May, having recommended the following gentlemen for admission into the navy as Assistant Surgeons:—1. James E. Lindsay, of North Carolina; 2. Henry F. McSherry, of Virginia; 3. John J. Gibson, of Illinois; 4. Osborn S. Inglehart, of Maryland; 5. Samuel J. Jones, of Pennsylvania; 6. Robert R. Gibbes, of South Carolina; 7. Joseph W. Shively, of Ohio.

Assistant Surgeons Daniel B. Conrad, James Laws, Francis L. Galt, John S. Kitchen, Albert L. Gihon, John Vansant, Edward R. Denby, and William M. Page, were examined and found qualified for promotion.

